# Design of Chitosan-Grafted Carbon Nanotubes: Evaluation of How the –OH Functional Group Affects Cs^+^ Adsorption

**DOI:** 10.3390/md13053116

**Published:** 2015-05-20

**Authors:** Shubin Yang, Dadong Shao, Xiangke Wang, Guangshun Hou, Masaaki Nagatsu, Xiaoli Tan, Xuemei Ren, Jitao Yu

**Affiliations:** 1 School of Environment and Chemical Engineering, North China Electric Power University, Beijing 102206, China; E-Mails: sdysb2006@163.com (S.Y.); xkwang@ipp.ac.cn (X.W.); tanxl@ipp.ac.cn (X.T.); renxm1985@163.com (X.R.); 2 Graduate School of Science and Technology, Shizuoka University, 3-5-1, Johoka-ku, Hamamatsu 432-8561, Japan; E-Mail: tmnagat@ipc.shizuoka.ac.jp; 3 School for Radiological and Interdisciplinary Sciences, Soochow University, Suzhou 215123, China; 4 Collaborative Innovation Center of Radiation Medicine of Jiangsu Higher Education Institutions, Suzhou 215123, China; 5 Institute of Resources & Environment, Henan Polytechnic University, Jiaozuo 454000, China; E-Mail: houguangshun@163.com

**Keywords:** carbon nanotube (CNTs), chitosan, Cs^+^ adsorption, –OH functional groups

## Abstract

In order to explore the effect of –OH functional groups in Cs^+^ adsorption, we herein used the low temperature plasma-induced grafting method to graft chitosan onto carbon nanotubes (denoted as CTS-g-CNTs), as raw-CNTs have few functional groups and chitosan has a large number of –OH functional groups. The synthesized CTS-g-CNT composites were characterized using different techniques. The effect of –OH functional groups in the Cs^+^ adsorption process was evaluated by comparison of the adsorption properties of raw-CNTs with and without grafting chitosan. The variation of environmental conditions such as pH and contact time was investigated. A comparison of contaminated seawater and simulated groundwater was also evaluated. The results indicated that: (1) the adsorption of Cs^+^ ions was strongly dependent on pH and the competitive cations; (2) for CNT-based material, the –OH functional groups have a positive effect on Cs^+^ removal; (3) simulated contaminated groundwater can be used to model contaminated seawater to evaluate the adsorption property of CNTs-based material. These results showed direct observational evidence on the effect of –OH functional groups for Cs^+^ adsorption. Our findings are important in providing future directions to design and to choose effective material to remedy the removal of radioactive cesium from contaminated groundwater and seawater, crucial for public health and the human social environment.

## 1. Introduction

Radioactive cesium is of serious social and environment concern as it readily dissolves in water, it has a high fission yield (6.09%), and a long half-life (T_1/2_ = 30.17 years) [1,2]. The major source of radioactive cesium is from the leaks of nuclear reactors, such as the nuclear disaster that occurred at Fukushima Daiichi in 2011 [3,4,5]. It is important to highlight that radioactive cesium can make its way into the food chain when present in wastewater, and do great harm to human health as well as to the living creatures in the aquatic environment [2,6,7]. Therefore, when accidentally released to the ground and sea, it is crucial for both the natural and the human social environment to find an effective material for removal of radioactive cesium from contaminated groundwater and seawater.

Over the past 50 years, various effective materials for capturing Cs^+^ ions have been developed. Datta *et al.* [1] designed a novel vanadosilicate with hexadeca-coordinated Cs^+^ ions as highly effective for Cs^+^ removal. Torad *et al.* [8] showed a large Cs^+^ adsorption capability of nano-structured Prussian blue particles. However, there are few reports about the effect of functional groups on Cs^+^ adsorption. In addition, the effects of functional groups and structure determine the direction for design and for choosing material for the uptake of radioactive cesium ions. Dwivedi *et al.* [9] considered that the high affinity of resorcinol-formaldehyde resin for Cs^+^ ions was attributed to the presence of the –OH group. In addition, the pH-dependence of Cs^+^ adsorption was usually attributed to the competition exchange of hydrogen in the –OH groups. However, to the best of our knowledge, direct observational evidence on the effect of the –OH functional group in Cs^+^ adsorption is still not available.

Carbon nanotubes (CNTs) and CNT-based materials gained widespread attention owing to their good chemical stability, relatively large specific area, and large average pore diameter [10,11,12]. Their size, shape, and physicochemical properties make them principal rivals for exploiting the growth of a potentially revolutionary material for diverse applications [13,14,15]. Conventional methods such as X-ray photoelectron spectroscopy (XPS), and X-ray powder diffraction (XRD) have been explicated to detect the structure and functional groups of CNTs. They reveal that regardless of the graphene structure, possession of few functional groups is the essential trait of pristine CNTs. Therefore, a significant amount of research activity into surface modification of CNTs was carried out to create functional groups on the nanotubes to explore their potential applications.

Conventional chemistry modification involving acid treatment and ultrasound may introduce wall damage of CNTs and cleave them into shorter pieces which is not especially environmentally friendly [16,17]. Low-temperature pressure plasma-induced grafting technique is an efficient method to graft functional groups onto CNT surfaces in the field of surface modifications and green eco-friendly chemistry [18]. Chitosan is one of the most abundant nontoxic biopolymers in nature with abundant hydroxyl groups. Diverse methods were applied to modify material by grafting chitosan to change its physical or chemical properties [2,19,20].

With these in mind, we herein designed novel chitosan grafted carbon nanotubes (CTS-g-CNTs) as Cs^+^ remover. The composite was synthesized by a radio frequency Ar-plasma-induced grafting method. The variance of environmental conditions such as pH, ionic strength, and adsorbent content was taken into account. We determined the effect of the hydroxyl group on the Cs^+^ adsorption process by comparison of the adsorption properties of CNTs with and without grafting chitosan. A comparison of contaminated seawater and simulated groundwater was also evaluated.

## 2. Results and Discussion

### 2.1. Material Characterization

**Figure 1:**
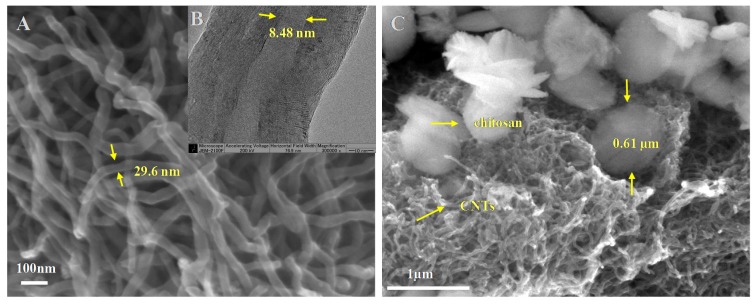
SEM (**A**) and TEM (**B**) images of raw-CNTs; and SEM image of CTS-g-CNTs (**C**).

The morphology and size of the raw-CNTs and CTS-g-CNTs were characterized by SEM and TEM. The CNTs (Figure 1A) have very smooth surfaces and the nanotubes are entangled, with a diameter of about 30 nm. From Figure 1B we can clearly observe the graphene sheet structure of raw-CNTs with an inner diameter of about 8.48 nm. However, the effective hydrated radius of cesium ions is only 0.33 nm, much smaller than the inner diameter of raw-CNTs [21,22,23]. Therefore, the cesium ions could easily diffuse into the inner part (pore) of the nanotubes, indicating that the pore filling is also one of the possible main mechanisms for the capture of Cs^+^ ions by CNT-based material. A large number of previous studies have emphasized the importance of physical adsorption in the adsorption mechanism of heavy metal ions [12,24,25]. For instance, Omura *et al.* [25] designed a size-controlled nanospace of hexacyanoferrate applied in trapping Cs^+^ ions. In Figure 1C, a more extensive three-dimensional network of the surface morphology between raw-CNTs and CTS-g-CNTs is observed. The CTS-g-CNTs depict large nanostructures of about 0.5–0.8 µm in diameter coated on the surface of CNTs. The obvious differences in the SEM images indicate that the CTS-g-CNTs composites have been synthesized successfully.

**Figure 2:**
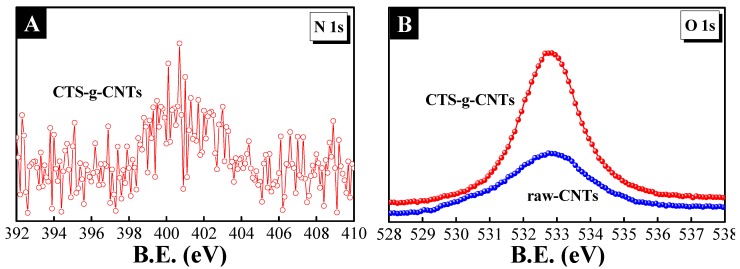
X-ray photoelectron spectroscopy (XPS) spectra of N 1s (**A**) and O 1s (**B**).

The surface properties of the samples were analyzed by XPS, which was used to ensure the elemental composition at the surface. The grafted chitosan was evidenced by the following XPS analysis. In our system, nitrogen only exists in chitosan; therefore, the nitrogen content can be an indication of the extent of surface coverage by chitosan [26]. Figure 2A illustrates the N 1s spectrum of CTS-g-CNTs, a nitrogen peak at 400.5 eV was observed, which was attributed to the amino groups of chitosan. Besides, obvious differences of the O 1s spectrum (Figure 2B) between raw-CNTs and CTS-g-CNTs were also observed. After chitosan coating, the O 1s (533 eV) peak intensity of CTS-g-CNTs significantly increased with regard to that of CNTs, which was attributed to the hydroxyl groups of chitosan.

**Figure 3:**
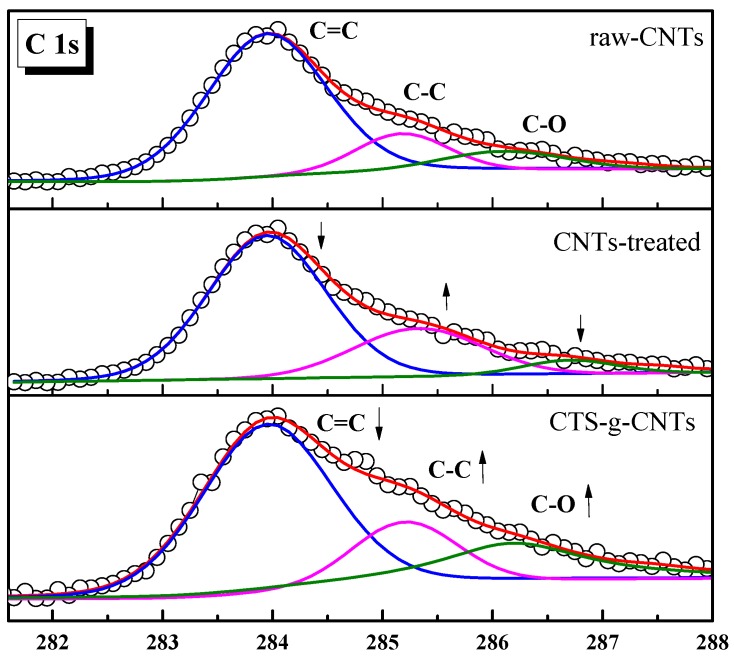
XPS spectra of C 1s.

In addition, the C 1s XPS spectra of raw-CNTs, plasma treated CNTs (denoted as: CNTs-treated) and CTS-g-CNTs are shown in Figure 3. The more detailed analysis of the XPS C 1s spectra is shown in Table 1. All of the C 1s XPS spectra have three characteristic peaks: C=C (284.4 eV), C–C (285.4 eV) and C–O (286.2 eV) peak. As can be seen from the quantitative analysis in Table 1, after plasma treated, the peak fraction of C=C decreases, whereas the peak fraction of C–C increases. Ar plasma can activate the C=C bonds of the CNTs surface to increase the reactivity of CNTs during the pre-treatment as previously reported [27,28,29]. These activated C=C bonds can interact with chitosan, resulting in a decrease in the peak fraction of C=C, whereas there is an increase in the fractions of C–C and C–O. These XPS analyses are valid indication of the effective connection of chitosan onto the CNTs structure. The CTS-g-CNT composites were synthesized successfully which was consistent with the results of SEM analysis.

**Figure 4:**
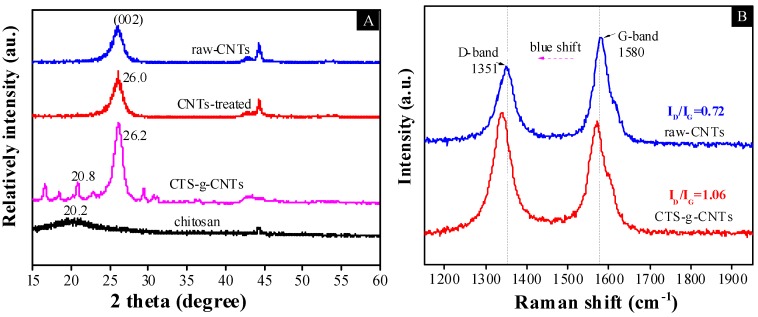
X-ray diffraction (XRD) patterns (**A**); and Raman spectra (**B**) of raw-CNTs and CNT-based materials.

X-ray diffraction (XRD) can provide useful information on the structural properties of CNT-based materials [17,30,31]. Figure 4A shows the XRD patterns of CNTs, CNTs-treated, CTS-g-CNTs and chitosan. For CNTs, a strong diffraction peak at *2*θ = 26.0° was observed, corresponding to the (002) planes of graphite. The peaks of the raw-CNT and CNTs-treated material are very intense and pointed, indicating a nanotube with excellent crystallinity. In addition, the XRD patterns of CNTs-treated and CNTs are very similar, meaning that there is only minor alteration in the structure of CNTs after Ar plasma treatment. The plasma treatment only activated the surface of CNTs without damaging the original orientation of CNTs alignment, as previously reported [27,32]. For CTS-g-CNTs, the peak at *2*θ = 26.2° is related to the characteristic of raw-CNTs. The dominant peak at *2*θ = 26.2° is still intense and sharp, indicating the CTS-g-CNTs composite shows a good crystallinity. Thus, the nanotube framework still shows strong influence on the properties of CTS-g-CNT composite. After loading with chitosan, many new miscellaneous and low intensity peaks appeared indicating lower crystallinity of the CTS-g-CNTs phase due to embedded chitosan. Namely, the CTS-g-CNTs composite had been synthesized. It was also observed that chitosan led to a small positive shift of the (002) plane of graphite, indicating that the distance between planes is reduced instead of increased. From Bragg’s Law [33,34], we can see that the chitosan addition does not have a significant impact on the mean distance between graphitic walls. The broad peak in the XRD pattern of chitosan indicates the amorphous state of chitosan. The narrow and weak peak at *2*θ = 20.8° in the XRD pattern of CTS-g-CNTs corresponds to the characteristics of chitosan. From the XRD studies it is observed that grafted chitosan affects both the intensity and peak position of the CNTs phase. Combined with these phenomena, it was proposed that they were related to a phase change in the structure of CNTs, in which chitosan had connected to the CNTs.

**Table 1:** Curve fitting results of X-ray photoelectron spectroscopy (XPS) C 1s spectra.

	C=C (%)	C–C (%)	C–O (%)
raw-CNTs	74.2	15.5	10.3
CNTs-treated	67.5	23.7	8.8
CTS-g-CNTs	60	17.8	22.2

Raman spectroscopy provides valuable information about the disorder in these materials. The Raman spectra of raw-CNTs and CTS-g-CNT composite are shown in Figure 4B. It is obvious that the spectrum of CNTs shows two characteristic bands at 1351 cm^−1^ (D-band) for the presence of defects (sp^3^ carbons, foreign atoms, *etc.*) of nanotubes and at 1580 cm^−1^ (G-band) for the tangential modes of CNTs. However, after chitosan had grafted, a blue shift of the characteristic peaks was observed. The slight shifts of the D-band and G-band could be related to some molecule embedding, which has been reported earlier [15,35]. The intensity relation between the two bands (*I*_G_/*I*_D_) is the most widely used tool to evaluate the grafting of carbon nanotubes [17,36]. According to the Raman data, the CTS-g-CNTs composite shows a higher *I*_G_/*I*_D_ ratio due to the defects created along the nanotube surface during the plasma-induced grafting process.

**Figure 5:**
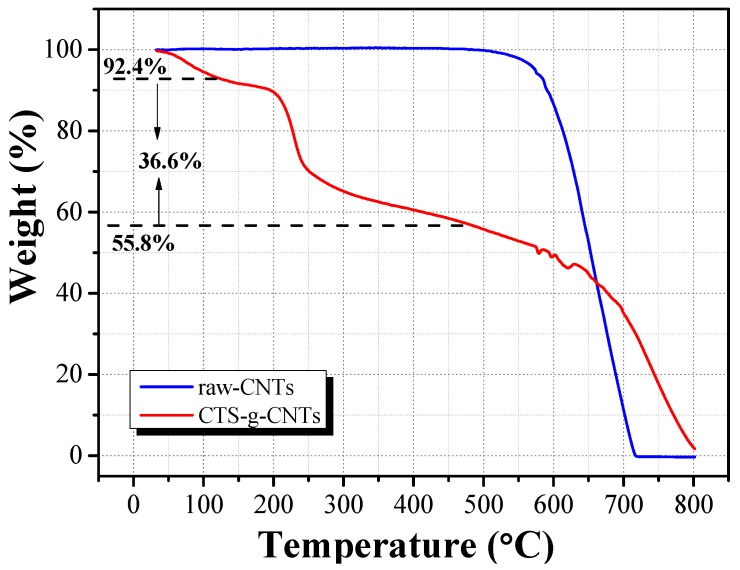
Thermogravimetric analysis (TGA) curves of raw-CNTs and CTS-g-CNT composite.

The weight percentage of grafted chitosan in CTS-g-CNTs composite can be estimated by thermogravimetric analysis (TGA). Figure 5 shows the TGA curves of CNTs and CTS-g-CNTs under 25% Ar/75% air atmosphere from 35 to 800 °C. For the TGA curve of CNTs, the weight loss of CNTs is negligible before 500 °C. For CTS-g-CNTs, there are three clearly separated weight loss stages in the range of 35–130 °C, 130–500 °C and 500–800 °C, which are mainly attributed to the combustion of the absorbed water, grafted chitosan, and CNTs, respectively. Assuming that the grafted chitosan was completely decomposed, the content of grafted chitosan was about 36.6% in the CTS-g-CNT composites.

### 2.2. Adsorption Experiment

**Figure 6:**
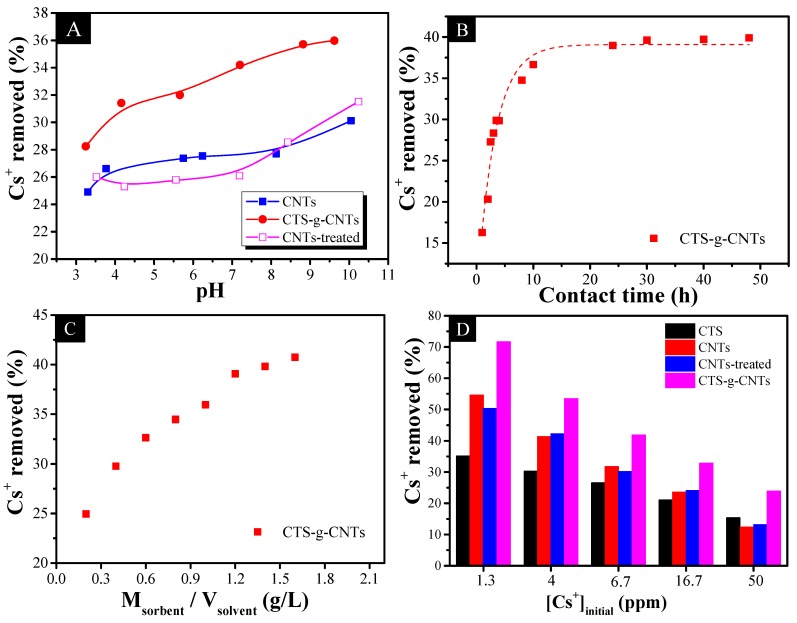
Effects of initial pH (**A**); contact time (**B**); sorbent content (**C**) and initial Cs^+^ concentration for Cs^+^ adsorption in contaminated simulated groundwater by CNTs-based materials with *m*_sorbent_/*V*_solvent_ = 0.6 g/L, [Cs^+^]_initial_ = 10.0 mg/L.

It has become an important point for researchers and enterprises to know how environmental conditions such as pH, contact time, sorbent content, *etc.*, influence Cs^+^ sorption capacity. Figure 6A shows how the pH influences Cs^+^ adsorption in contaminated simulated groundwater using 0.6 g/L adsorbents. It is noted that the removal percentage of Cs^+^ ions depends on pH value. We considered that there are two main reasons for the effect of pH value. The first is the surface properties of materials involving the surface charge of material and the protonation state of functional groups on the materials. At high pH, the material should possess a negative charge and more deprotonated functional groups, resulting in more Cs^+^ ions being adsorbed through electrostatic interaction. The second one is the fierce competition between hydronium ions (H_3_O^+^, 0.28 nm) and hydrated cesium ions (0.33 nm) [37,38]. At high pH, it is expected that low concentration of H_3_O^+^ will compete for the adsorption sites with hydrated cesium ions. As can be seen from the effect of pH in Figure 6A, the difference of the Cs^+^ removal percentage onto CNTs or CNT-treated material is negligible (<2.0%). Therefore, the plasma treatment cannot improve the adsorption capacity of CNTs in the removal of Cs^+^ ions. By comparison with raw-CNTs, the adsorption capacity of CTS-g-CNTs is much higher by 11%–24%. We should note that the adsorption of Cs^+^ ions on CTS-g-CNTs at pH >6.5 increases more quickly than that of Cs^+^ on raw-CNTs. This is due to the increased –OH functional groups which are closely related to the grafted chitosan. With the increase of pH, more –OH functional groups could react with Cs^+^ ions, leading to a rapid increase of the adsorption percentage. Therefore, for CNT-based material, –OH functional groups have a positive effect on Cs^+^ removal.

The effect of contact time in the removal of Cs^+^ from contaminated simulated groundwater by 0.6 g/L CTS-g-CNTs at pH = 7 was detected (Figure 6B). It was noted that the adsorption of Cs^+^ reached equilibrium in about 10 h. Therefore, the shaking time (contact time) of 24 hours is sufficient to reach full equilibrium state. The adsorption of Cs^+^ by 0.6 g/L CTS-g-CNTs at pH = 7 as a function of sorbent content is shown in Figure 6C. It is expected that the removal of Cs^+^ ions increases with increasing solid content; the more solid content the more active efficient sites for Cs^+^ ions.

The comparison of the degree of Cs^+^ removal for CTS, CNTs, CNTs-treated and CTS-g-CNTs sorbents at 1.3 ppm ≤ Cs^+^ ≤ 50 ppm, *m*_sorbent_/*V*_solvent_ = 0.6 g/L, and pH = 7 is shown in Figure 6D. The removal efficiency of the different sorbents was in the order of CTS < CNTs ≈ CNTs-treated < CTS-g-CNTs. Obviously, there is almost no difference between CNTs and CNT-treated while a clear increase was shown in CTS-g-CNT. We consider that there are two reasons for the enhanced efficiency of CTS-g-CNT. Dwivedi *et al.* [9] reported the high affinity of resorcinol-formaldehyde resin for Cs^+^ ions to be attributed to the presence of the –OH group. In addition, chitosan is a nontoxic biopolymer with abundant hydroxyl groups. Therefore, we consider that the improved efficiency of CTS-g-CNTs is largely due to the increased –OH functional groups which are closely related to the grafted chitosan. The low removal efficiency of CTS is related to the peculiar structure of chitosan which appears lamellar when mixed with CNTs, while it is amorphous when considered alone in XRD patterns.

In addition, chitosan is a nontoxic biopolymer with abundant hydroxyl groups. Therefore, we consider that the improved efficiency of CTS-g-CNTs is largely due to the increased –OH functional groups which are closely related to the grafted chitosan.

**Figure 7:**
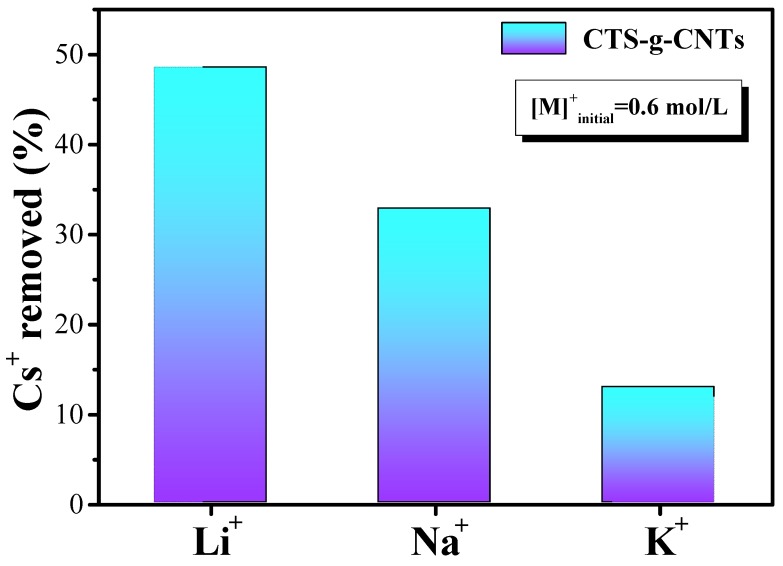
Comparison of the removal percentage of Cs^+^ ions obtained using CTS-g-CNTs in presence of competitive cations with *m*_sorbent_/*V*_solvent_ = 0.6 g/L and [Cs^+^]_initial_ = 10.0 mg/L.

An additional important characteristic of Cs^+^ sorption is competitive cation dependence (Figure 7). Many researches have shown that ion exchange is an important principle in Cs^+^ adsorption process. We can see that the adsorption of Cs^+^ by CTS-g-CNTs in Figure 7 is strongly dependent on the competitive cations. In the presence of different competitive cations, the removal percentage of Cs^+^ ions decreased in the order of Li^+^ (48.5%) > Na^+^ (32.8%) > K^+^ (12.9%). This is closely related to the hydrated radii of cesium and the competitive cations. The hydrated radii of cations [23,39,40,41] decreased in the order of Li^+^ (0.38 nm) > Na^+^ (0.36 nm) > K^+^ (0.33 nm) ≈ Cs^+^ (0.33 nm), therefore, K^+^ ions are the most important inhibitors of Cs^+^ adsorption.

In addition, the distribution coefficient (*K_d_*) of Cs^+^ in the presence of competitive cations and for various materials collected from the recent references is listed in Table 2. It can be seen that the *K_d_* value substantially decreased in the same order of Li^+^ > Na^+^ > K^+^. In addition, the *K_d_* value expresses the chemical binding affinity of Cs^+^ ion to the sorbents, which is helpful for us in understanding the relative efficiency of the materials. It is obvious that the CTS-g-CNTs composite shows a bigger distribution coefficient than the other sorbents, indicating the CTS-g-CNTs composite shows a higher adsorption capacity (Table 2). Therefore, the CTS-g-CNTs composite could be a good candidate for the remediation of radioactive cesium nuclear waste water.

**Table 2:** Comparison of *K*_d_ values obtained using different materials in the presence of competitive cations.

Materials	Competitive Cations	*K*_d_ (mL/g)	References
CTS-g-CNTs	0.1 M Li^+^	152.8	This work
	0.1 M Na^+^	118.6	
	0.1 M K^+^	94.7	
CA	3.5 mM Na^+^	69.8	[ 42]
	2.1 mM K^+^	66.5	
IA	3.5 mM Na^+^	43.2	[ 42]
	2.1 mM K^+^	26.6	
PB-coated MNP	0.1 M Na^+^	56.4	[ 43]
	0.1 M Mg^2+^	112.5	
	0.1 M K^+^	14.3	

**Figure 8:**
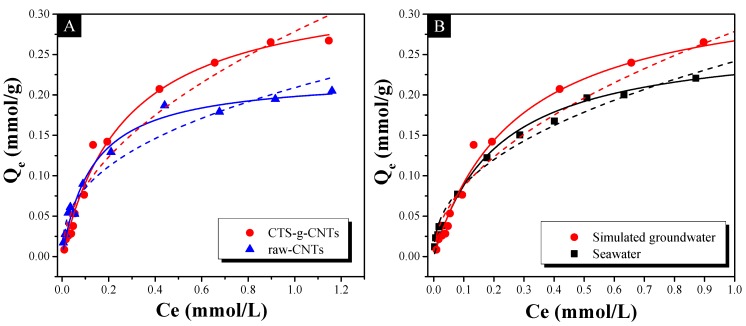
Adsorption isotherms of Cs^+^ on CTS-g-CNTs and raw-CNTs in simulated groundwater (**A**) and adsorption isotherms of Cs^+^ on CTS-g-CNTs in contaminated stimulated groundwater and seawater (**B**) with pH = 7.0 and *m*_sorbent_/*V*_solvent_ = 0.6 g/L. Symbols denote experimental data, solid lines represent model fitting of the Langmuir equation, and dash lines represent the model fitting of the Freundlich equation.

Two comparisons of Cs^+^ adsorption isotherms are shown in Figure 8. We used the Langmuir [37,44] and Freundlich [45] isotherm models, both of which are the most widely used ones among the abundant isotherm models, to fit the experimental data in order to understand the adsorption mechanism. The Langmuir (1) and Freundlich (2) equations are expressed as follow:

(1) $$Q_{\text{e}}=\frac{Q_{\max}K_{L}C_{e}}{1+K_{L}C_{e}}$$

(2) $$Q_{e}=K_{\text{F}}C_{e}^{1/n}$$

where *Q*_e_ is the equilibrated cesium ion concentration, *Q*_max_ (mg/g) is the maximum sorption capacity, *K*_L_ (L/mg) is the Langmuir adsorption constant and 1/*n* is the Freundlich adsorption constant.

The related parameters of the two models are listed in Table 3. From the correlation coefficient (*R*^2^), the Langmuir model fits the experimental data better than the Freundlich model. The first comparison (Figure 8A) is between the raw-CNTs and CTS-g-CNTs for Cs^+^ adsorption capacity, as it is very useful to understand the effect of –OH functional groups. The maximum adsorption capacity (*Q*_max_) of Cs^+^ on CTS-g-CNTs is 0.340 mmol/g, and much higher than that of Cs^+^ on raw-CNTs (0.224 mmol/g). Therefore, after having added many –OH functional groups, which are derived from the grafted chitosan, the adsorption capacity of CNTs is enhanced by about 52%. In a word, the effect of –OH functional groups in our system is positive, which is consistent with results showed in Figure 6A. The second comparison (Figure 8B) is between the contaminated seawater and simulated groundwater for Cs^+^ adsorption capacity by CTS-g-CNTs. The purposes of this comparison were to detect the stability of CTS-g-CNTs in seawater and to evaluate the simulated groundwater. The adsorption isotherm in contaminated seawater is similar to that in contaminated simulated groundwater, indicating that the CTS-g-CNTs could remain stable in seawater. In addition, the *Q*_max_ for Cs^+^ ions by CTS-g-CNTs in contaminated simulated groundwater (0.340 mmol/g) is a bit larger than that of Cs^+^ ions in contaminated seawater (0.272 mmol/g), which is attributed to the higher concentration of competitive cations in seawater. Therefore, to a certain extent, we can consider that the simulated groundwater can be used to model the seawater in our system.

**Table 3:** Sorption constants for Langmuir and Freundlich isotherm models.

System	Sorbent	Langmuir			Freundlich		
		*Q*_max_ (mmol/g)	*K*_L_ (L/mmol)	*R*^2^	*n*	*K*_F_ (mmol/g)	*R*^2^
Simulated groundwater	raw-CNTs	0.224	7.62	0.976	2.55	0.209	0.951
	CTS-g-CNTs	0.340	3.67	0.988	1.97	0.279	0.944
Seawater	CTS-g-CNTs	0.272	4.79	0.987	2.30	0.242	0.986

## 3. Experimental Section

### 3.1. Materials

The CNTs used in this work are multi-walled CNTs, and were prepared by chemical vapor deposition as previously reported [27,36]. Chitosan 100, cesium chloride, sodium hydroxide, and other reagents were purchased from Wako Pure Chemical Industries, Ltd. (Osaka, Japan). All chemicals used were analytical grade and the solutions were prepared with Milli-Q water. The seawater (Cl^−^: 19,400 ppm, Na^+^: 10,800 ppm, Mg^2+^: 1270 ppm, Ca^2+^: 412 ppm, K^+^: 392 ppm, pH = 7.6) used in this study was taken from the Pacific Ocean near Hamamatsu city, which is in the eastern coast of Japan. The simulated groundwater was prepared by Na^+^: 230 ppm, Mg^2+^: 240 ppm, Li^+^: 70 ppm, K^+^: 390 ppm, pH = 5.0.

### 3.2. Synthesis of CTS-g-CNTs

The CTS-*g*-CNT composites were synthesized by the plasma-induced grafting method involving surface activation by radio frequency Ar plasma and chitosan grafting procedures. An amount of 0.1 g of raw-CNTs was firstly pre-treated by Ar plasma at a pressure of 50 Pa. The treatment time was for 10 min and the plasma power was 80 W. Then 150 mL 1.0% *w*/*v* of chitosan solution prepared in 1% *v*/*v* acetic acid was immediately injected into the plasma treated CNTs. The mixture was heated to 80 °C and the temperature maintained with fast stirring for 24 h. The CTS-g-CNT composites were collected by centrifuging, washed, dried, and analyzed by SEM (JSM-7001F, JEOL, Tokyo, Japan), XRD equipped with Cu Kα radiation (λ = 0.154 nm), LabRam HR Raman spectrometry, XPS (ESCA-3400, Shimadzu, Kyoto, Japan) with Mg Kα X-ray source and TGA (DTG-60A, Shimadzu, Kyoto, Japan) under 25% Ar/75% air atmosphere with a heating rate of 10 °C/min from 35 to 800 °C.

**Figure 9:**
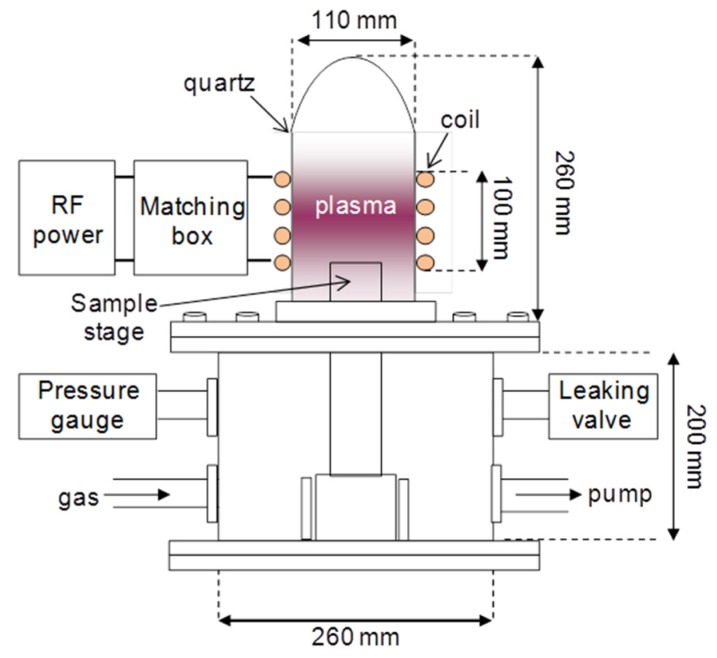
Schematic view of the experimental setup for inductively coupled radio frequency plasma.

**Figure 10:**
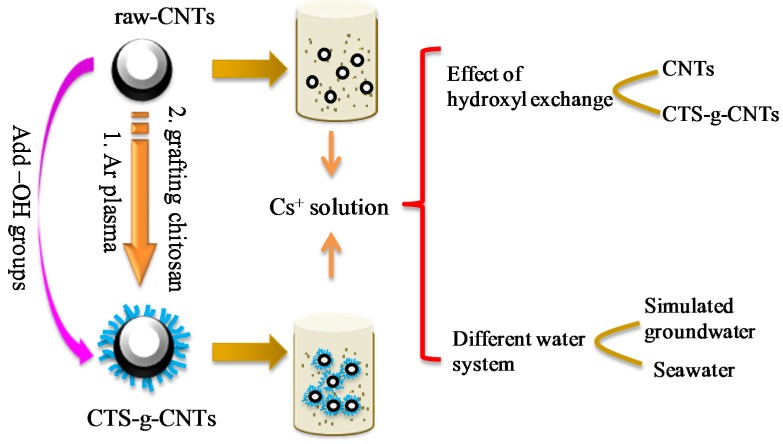
Schematic illustration of the designed research approach.

The schematic view of the inductively-coupled radio frequency plasma device is described in Figure 9. The schematic of the formation of CTS-g-CNTs and the schematic illustration of the research approach are shown in Figure 10.

### 3.3. Cesium Adsorption Experiment

An amount of 6 mg of CTS-g-CNT composites was added to the simulated groundwater or seawater, then a dilute CsCl solution with different amounts of Cs^+^ was added into the above mixture solution to control the solution volume of 6 mL, and the concentration of Cs^+^ ranging from 1.0 to 42 ppm. The desired pH was adjusted by adding small volumes of HCl or NaOH (0.01 or 0.1 mol/L). The suspension was then shaken at ambient conditions. The shaking time was fixed at 24 h to ensure that the adsorption could achieve full equilibrium. Finally, the solid and liquid phases were separated by centrifuging at 14,000 rpm for 30 min, and the concentration of Cs^+^ in solution was determined by atomic absorption spectroscopy.

The distribution coefficient (*K*_d_, mL/g) and the percentage of Cs^+^ removed by the adsorbents were calculated according to the following equations:

(3) $$K_{\text{d}}=\frac{(C_{o}-C_{t})V}{132.9W}$$

(4) $${\text{Cs}}^{+}\;removed(\%)=\frac{\left(C_{o}-C_{t}\right)}{C_{o}}\times 100\%$$

where *C*_o_ is the initial concentration of Cs^+^ (mg/L), *C_t_* represents the concentration of Cs^+^ at time *t*, *V* represents the total volume of the solution (L), *W* is the mass of adsorbent (g) and 132.9 is the standard atomic weight of cesium.

## 4. Conclusions

To summarize, we synthesized CTS-g-CNT composites by the plasma-induced grafting method to explore the effect of the –OH functional groups in Cs^+^ adsorption. The variation of environmental conditions such as pH, contact time, *etc.* were taken into account. A comparison of contaminated seawater and simulated groundwater was also performed. The adsorption of Cs^+^ ions was strongly dependent on pH and the competitive cations. By examining the materials and the two aqueous solution systems, one can see that: (1) modification with many –OH functional groups can increase the adsorption capacity for Cs^+^ ions to a certain extent; (2) we can use the simulated groundwater to model the contaminated seawater to evaluate the adsorption property of CNTs-based material. These results showed the direct observational evidence on the effect of the –OH functional group for Cs^+^ adsorption. Our findings are important to provide future directions to design and choose effective materials to remedy the removal of radioactive cesium from contaminated groundwater and seawater, which is crucial for both the natural environment and the human social environment.
